# Vegetative Hyphal Fusion and Subsequent Nuclear Behavior in *Epichloë* Grass Endophytes

**DOI:** 10.1371/journal.pone.0121875

**Published:** 2015-04-02

**Authors:** Jun-ya Shoji, Nikki D. Charlton, Mihwa Yi, Carolyn A. Young, Kelly D. Craven

**Affiliations:** 1 The Samuel Roberts Noble Foundation, Plant Biology Division, 2510 Sam Noble Parkway, Ardmore, Oklahoma 73401, United States of America; 2 The Samuel Roberts Noble Foundation, Forage Improvement Division, 2510 Sam Noble Parkway, Ardmore, Oklahoma 73401, United States of America; Friedrich Schiller University, GERMANY

## Abstract

*Epichloë* species (including the former genus *Neotyphodium*) are fungal symbionts of many agronomically important forage grasses, and provide their grass hosts with protection from a wide range of biotic and abiotic stresses. *Epichloë* species include many interspecific hybrids with allodiploid-like genomes, which may provide the potential for combined traits or recombination to generate new traits. Though circumstantial evidence suggests that such interspecific hybrids might have arisen from nuclear fusion events following vegetative hyphal fusion between different *Epichloë* strains, this hypothesis has not been addressed empirically. Here, we investigated vegetative hyphal fusion and subsequent nuclear behavior in *Epichloë* species. A majority of *Epichloë* strains, especially those having a sexual stage, underwent self vegetative hyphal fusion. Vegetative fusion also occurred between two hyphae from different *Epichloë* strains. Though *Epichloë* spp. are uninucleate fungi, hyphal fusion resulted in two nuclei stably sharing the same cytoplasm, which might ultimately lead to nuclear fusion. In addition, protoplast fusion experiments gave rise to uninucleate putative hybrids, which apparently had two markers, one from each parent within the same nucleus. These results are consistent with the notion that interspecific hybrids arise from vegetative hyphal fusion. However, we also discuss additional factors, such as post-hybridization selection, that may be important to explain the recognized prevalence of hybrids in *Epichloë* species.

## Introduction

Phylogeny, the evolutionary paths that gave rise to modern life, is expressed as phylogenetic trees, representing the vertical transmission of genes from a parent to offspring. However, it is now clear that genetic material can also be transferred horizontally between two organisms, accounting for the acquisition of many adaptively important genetic traits [1], [2].

In fungi, genetic material may be horizontally transferred in the form of a gene(s), an entire chromosome [3], [4], or even as complete chromosomal sets [5–8]. Such transfers can occur within a species and even between organisms of broader taxonomic separation across mating barriers. As a consequence, horizontal transfer of genetic material is a significant driver of fungal diversity, with implications for both agronomy and industry. For example, horizontal gene/chromosome transfer has been documented as the likely mechanism for the emergence of several new fungal phytopathogens [3], [5], [6], [9–12]. Furthermore, a majority of yeasts utilized in the wine and beer industries [7], [8], as well as agronomically important grass endophytes of the genus *Epichloë* [6], [13], are either natural or induced allopolyploid-like interspecific hybrids whose genomes are mosaics or combinations of two or more parental chromosomal sets.

The mechanism underlying horizontal transfer of genetic material in fungi remains largely unclear despite its importance in both natural and directed evolution of microbes for phytopathology [3], [12] and strain improvement [7]. In yeast, interspecific hybrids arise sexually from rare mating between two species [7], [14]. In contrast, horizontal transfer of genetic material in filamentous fungi is suspected to occur vegetatively through hyphal fusion [3], [4], [6], [15].

Vegetative hyphal fusion (VHF), or anastomosis, is a process commonly found in filamentous fungi that links neighboring hyphae within a mycelium to facilitate distribution of water, nutrients, and signaling molecules across the colony [16–18]. In addition to “self-fusion” that links hyphae within the same mycelium, VHF can also occur between hyphae from two genetically different fungal individuals. Such non-self fusion generally triggers the vegetative incompatibility response that leads to death of the fused cell [19]. However, fused cells may occasionally survive when the vegetative incompatibility response is suppressed [20], or when the fungus lacks this response, as in the case of *Epichloë* [21]. When the fused cell survives, two types of nuclei, one from each fungal individual share the same cytoplasm, which in turn may lead to horizontal transfer of genetic material when followed by fusion of the two nuclei (i.e., karyogamy; [6], [22]), or transfer of genes/chromosomes from one nucleus to the other [3], [4]. VHF may also be important for sexual reproduction, since mutant strains lacking this ability are often sterile [17].

*Epichloë* species (including the former genus *Neotyphodium* [23]) are systemic symbionts of cool-season grasses, which include agronomically important sources of forage [24], [25]. These symbionts reside in the intercellular spaces of foliar tissues of the host plant [26], [27], and typically provide the host with protection from a variety of biotic and abiotic stresses [28–32] in exchange for nutrients. Though some of these protective traits are beneficial in agronomy (e.g., drought tolerance and toxicity to insect herbivores), others are not (e.g., toxicity to grazing mammalian herbivores). Therefore, the generation or discovery of new endophyte strains with desirable traits is an important goal for the agronomic application of *Epichloë* species. Many of the most useful *Epichloë* strains currently recognized are interspecific hybrids with allodiploid (or sometimes even allotriploid) origins [13], [24], [25], [33]-[37]. It has been suggested that such hybrid endophytes may have beneficial attributes inherited from each of its progenitors [6], [38], [39], and thus, generation of new hybrids may be a sound option to obtain new *Epichloë* strains with novel sets of beneficial traits. For this reason, we were interested in studying VHF in *Epichloë* species in relation to its potential role in the generation of hybrids. Previous studies on VHF in *Epichloë* species focused on its genetic basis and its role in colony development and the establishment of symbiosis [40–43]. In this study, our focus was on the potential role of VHF in emergence of interspecific hybrids, which we investigated directly through cytological analysis of fates of nuclei and other organelles after cell fusion, in addition to comparing occurrence of VHF in different *Epichloë* species.

## Materials and Methods

### Plasmid construction

Plasmids used in this study are summarized in Table 1. Plasmid pYH2A is a kind gift from Dr. Ines Engh and Prof. Ulrich Kück at the University of Bochum, and encodes *Sordaria macrospora* histone H2A fused to enhanced yellow fluorescent protein (EYFP) under the control of the *Aspergillus nidulans gpdA* promoter and *trpC* terminator [44]. It also contains the hygromycin resistance gene *hph* expressed under the control of the *A*. *nidulans trpC* promoter as a selection marker. The plasmids pAL1 [45] and pAL10-Lifeact [46] containing sGFP and TagRFP, respectively, are kind gifts from Dr. Alexander Lichius and Prof. Nick D. Read at the University of Manchester.

**Table 1:** Plasmids used in this study.

Plasmid	Genes	Source
pYH2A	P*gpdA-h2a-eyfp-*T*trpC* P*trpC-hph*	[44]
pAL1	P*ccg-1-sgfp-*T*trpC* P*trpC-bar*	[45]
pAL10-Lifeact	P*tef-1-lifeact-tagrfp*-T*trpC* P*trpC-nat1*	[46]
pEfso-Comp	P*sftA-sftA-*T*sftA* P*trpC-nptII*	[40]
pYGFP	P*gpdA-sgfp-*T*trpC* P*trpC-hph*	This study
pYtRFP	P*gpdA-tagrfp-*T*trpC* P*trpC-hph*	This study
pYtRFP-Gen	P*gpdA-tagrfp-*T*trpC* P*trpC-nptII*	This study
pYHG	P*gpdA-h2a-sgfp-*T*trpC* P*trpC-hph*	This study
pYHR-Gen	P*gpdA-h2a-tagrfp-*T*trpC* P*trpC-nptII*	This study

To generate plasmids for visualizing nuclei with green (GFP) or red (TagRFP) fluorescent proteins, the EYFP-encoding gene in pYH2A was replaced by GFP- or TagRFP-encoding genes as follows. The *gfp* gene was amplified using primers IF-gpd-sGFP-fw and IF-sGFP-TTrpC-rv (Table 2) with a pAL1-derived construct as a template. The *tagrfp* gene was amplified using primers IF-gpd-tRFP-fw and IF-tRFP-TTrpC-rv with pAL10-Lifeact as a template. The *h2a-eyfp* fusion gene in pYH2A was replaced with the amplified *gfp* or *tagrfp* genes through double digestion by *Nco* I and *Not* I restriction enzymes (New England Biolabs, Ipswitch, MA, USA) followed by *in vitro* recombination using the In-Fusion HD Cloning System (Clontech, Mountain View, CA, USA), resulting in pYGFP and pYtRFP plasmids (Table 1). To replace the hygromycin resistance gene *hph* in pYtRFP with the geneticin resistance gene *nptII* [47], *nptII* in the plasmid pEfso-Comp [40] was amplified using primers IF-PtrpC-Gen-fw and IF-Gen-TtrpC-rv-2. The amplified fragment was inserted through *in vitro* recombination into pYtRFP double-digested by *Afl* II and *Apa* I (New England Biolabs), resulting in the pYtRFP-Gen plasmid. The *h2a* gene, amplified with primers IF-PtrpC-H2A-fw and either IF-H2A-GFP-rv or IF-H2A-tRFP-rv with pYH2A as a template, was then reinserted into pYGFP or pYtRFP-Gen plasmids through digestion by *Nco* I followed by *in vitro* recombination. This resulted in plasmid pYHG encoding a histone H2A-GFP fusion protein and harboring the hygromycin resistance gene, and pYHR-Gen encoding a histone H2A-TagRFP fusion protein and harboring the geneticin resistance gene (Table 1). The gene sequence of all PCR-amplified inserts was checked by DNA sequencing. Primer sequences are summarized in Table 2.

**Table 2:** Primers used for this study.

Primer	Primer sequence (5' to 3')	Target gene	Restriction sites
IF-gpd-sGFP-fw	**GCAGACATCA****CC*ATGG****TGAGCAAGGGCGAGGA*	*gfp*	*Nco* I
IF-sGFP-TTrpC-rv	**AGTTCTAGA****GCGGCC**GC*TTACTTGTACAGCTCGTCCATGC*	*gfp*	*Not* I
IF-gpd-tRFP-fw	**GCAGACATCA****CC*ATGG****TGTCTAAGGGCGAAGAGC*	*tagrfp*	*Nco* I
IF-tRFP-TTrpC-rv	**AGTTCTAGA****GCGGCC**GC*TTACTTGTACAGCTCGTCCATGC*	*tagrfp*	*Not* I
IF-PtrpC-Gen-fw	**GTCATACCTT*****CTTAA****G**TTCGCCCTTCCTC*	*nptII*	*Afl* II
IF-Gen-TtrpC-rv-2	**TTGGTTTAGGGTTA****G**GGCCC*GTCTCTTGACGACCGTTGAT*	*nptII*	*Apa* I
IF-PtrpC-H2A-fw	***GCAGACATCA******CCATG****G**CT*	*h2a*	*Nco* I
IF-H2A-GFP-rv	***CCCTTGCTCA******CCATG****G**C*	*h2a*	*Nco* I
IF-H2A-tRFP-rv	**CCCTTAGA*CA******CCATG****G**CAGTCTTGC*	*h2a*	*Nco* I

Bold letters indicate vector-derived sequences for *in vitro* recombination.

Underline indicates restriction sites.

Italic letters indicate template DNA-derived sequences for PCR.

### Fungal strains and cultures

*Epichloë* strains (Table 3) were obtained from Prof. Christopher L. Schardl at the University of Kentucky, or from ATCC (http://www.atcc.org). Cultures were maintained on potato dextrose agar (PDA; Becton, Dickinson and Company, Sparks, MD, USA) plates at 25°C. Transformants harboring pYHG or pYHR-Gen plasmids were maintained on PDA containing 150 μg/mL hygromycin B (Omega Scientific, Inc., Tarzana, CA, USA) or 150 μg/mL geneticin (G418 sulfate from Mediatech, Manassas, VA, USA), respectively. Putative hybrid *Epichloë* were maintained on PDA containing 150 μg/mL hygromycin B and 200 μg/mL geneticin. For microscopy, *Epichloë* strains were grown on either 10- or 100-times-diluted potato dextrose broth (PDB; Becton, Dickinson and Company) supplemented with 2% agar (hereafter referred to as diluted PDA) for sparse mycelial growth to allow visualization of individual hyphae.

**Table 3:** Vegetative hyphal fusion in *Epichloë* grown in culture.

species^1^	strain	ATCC (CBS) #	sexual status	hybrid status^2^	hyphal fusion^3^	hyphal cords^4^	Reference
*Epichloë amarillans*	E52	200743	sexual	NH	++	++	[35]
*Epichloë amarillans*	E57	200744	sexual	NH	++	++	[35]
*Epichloë baconii*	357/9039-1	200745	sexual	NH	++	++	[35]
*Epichloë baconii*	424/9270	200746	sexual	NH	++	++	[35]
*Epichloë brachyelytri*	E1040	200752	sexual	NH	++	++	[86]
*Epichloë brachyelytri*	E1045	200753	sexual	NH	++	++	[86]
*Epichloë brachyelytri*	E1124	201560	sexual	NH	++	++	[86]
*Epichloë bromicola*	501/9053 [9053]	200749 [CBS 100091, E501]	sexual	NH	++	++	[87]
*Epichloë bromicola*	E799	201559	sexual	NH	++	++	[87]
*Epichloë elymi*	E56	201551	sexual	NH	ND	+	[88]
*Epichloë elymi*	E757	201553	sexual	NH	+	+	[86]
*Epichloë elymi*	E184	200850	sexual	NH	++	++	[88]
*Epichloë festucae*	E2368		sexual	NH	++	++	[89]
*Epichloë festucae*	Fl1	MYA-3407	sexual	NH	++	++	[27]
*Epichloë festucae*	E434 [9141]	MYA-434	sexual	NH	++	++	[90]
*Epichloë festucae*	E1035.33	MYA-446	sexual	NH	++	-	
*Epichloë festucae*	E189	90661	sexual	NH	++	++	[88]
*Epichloë glyceriae*	E2772	200755	sexual	NH	++	++	[86]
*Epichloë glyceriae*	E277/8734	200747	sexual	NH	+	+	[86]
*Epichloë sylvatica*	354	200748 [CBS 100089, E354]	sexual	NH	+	++	[87]
*Epichloë sylvatica*	503/9301-1 [9301]	200751 [CBS 10086, E503]	sexual	NH	++	++	[87]
*Epichloë typhina*	E8	200736	sexual	NH	++	++	[88]
*Epichloë typhina*	505/9410	200739	sexual	NH	+	-	[35]
*Epichloë typhina*	E1022/9515	201668	sexual	NH	++	++	[35]
*Epichloë typhina* subsp. *clarkii*	426/9342	200741	sexual	NH	++	++	[90]
*Epichloë typhina* subsp. *clarkii*	Holcus 3	90168	sexual	NH	++	++	[91]
*Epichloë festucae* var. *lolii*	e135		asexual	NH	ND	+	[35]
*Epichloë festucae* var. *lolii*	e136		asexual	NH	+	++	
*Epichloë festucae* var. *lolii*	e137		asexual	NH	ND	-	
*Epichloë festucae* var. *lolii*	PRG1		asexual	NH	++	+	Young et al., unpublished
*Epichloë festucae* var. *lolii*	PRG3		asexual	NH	+	+	Young et al., unpublished
*Epichloë festucae* var. *lolii*	PRG15		asexual	NH	+	++	Young et al., unpublished
*Epichloë coenophiala*	e19	90664	asexual	H	ND	++	[92]
*Epichloë coenophiala*	93030		asexual	H	+	++	
*Epichloë coenophiala*	NFe45132 [GK45132]		asexual	H	ND	+	[93]
*Epichloë coenophiala*	NFe45078 [GK45078]		asexual	H	++	++	[93]
*Epichloë coenophiala*	NFe45118 [GK45118]		asexual	H	ND	++	[93]
*Epichloë Canadensis*	NFe692 [CWR5]		asexual	H	++	-	[78]
*Epichloë Canadensis*	NFe726 [CWR34]		asexual	H	++	-	[78]
*Epichloë* sp. FaTG-2	NFTF1800		asexual	H	++	-	
*Epichloë* sp. FaTG-2	NFe45081 [GK45081]		asexual	H	++	+	[93]
*Epichloë* sp. FaTG-2	NFe45103 [GK45103]		asexual	H	++	++	[93]
*Epichloë* sp. FaTG-3	NFe1100		asexual	H	ND	-	[39]
*Epichloë* sp. PauTG-1	e55		asexual	H	+	+	[35]

1) Species names are based on the revised nomenclature of *Epichloë* species [23].

2) NH; non-hybrid. H; hybrid.

3) Hyphal fusion

++, commonly undergoing vegetative hyphal fusion (the average number of vegetative hyphal fusion in the observed area in one set of experiments (8067.6 μm^2^) was ≥ 1, which corresponds to ca. > 10 vegetative hyphal fusion per mm^2^)

+, rarely undergoing vegetative hyphal fusion (the average number of vegetative hyphal fusion in the observed area was < 1)

ND, fusion not detected

4) Hyphal cords (see S2 Fig);

++, extensive cords of more than ten hyphae running side by side

+ minor cords of ca. five hyphae

-, no cords

### Subcellular staining

Cell walls, mitochondria, and vacuoles were stained with 25 μM Calcofluor White (CFW; Sigma-Aldrich, St. Louis, MO, USA), 1 μM MitoTracker Red CM-H_2_XRos (Life Technologies, Grand Island, NY, USA), and 20 μM Oregon Green 488 carboxylic acid diacetate (cDFFDA; Life Technologies), respectively. An agar block containing mycelia was placed upside down in phosphate-buffered saline (PBS; 8.0 g NaCl, 0.2 g KCl, 1.44 g Na_2_HPO_4_, 0.24 g KH_2_PO_4_ per liter [pH 7.4]) containing a fluorescent dye(s), which was mounted on a 48- by 65-mm cover glass (Thomas Scientific, Swedesboro, NJ, USA). After 30 min of incubation at 25°C, the agar block was washed twice with PBS and subjected to microscopy. For staining of vacuoles in *E*. *festucae* E2368 residing in the tall fescue plant, epidermal layers of leaf sheath from the host tiller were incubated in 500 mM sodium citrate buffer (pH 3.7) with 20 μM cDFFDA for 30 min, washed twice in PBS, and subjected to microscopy. Aniline blue and Alexa Fluor 488-conjugated wheat germ agglutinin (WGA-AF) staining was performed as described previously [48].

### Confocal microscopy

Confocal microscopy was performed with a Leica TCS SP2 AOBS confocal laser-scanning microscope (Leica Microsystems, Buffalo Grove, IL) with HC PL APO 20× (NA 0.7), HCX PL APO 60× (NA 1.2), and HCX PL APO 100× (NA 1.4) objective lenses (Leica Microsystems). CFW and aniline blue fluorescence was recorded by excitation with a 405-nm blue diode laser and detection of emission fluorescence between wavelengths of 415 and 520 nm. GFP, cDFFDA, and Alexa Fluor 488 fluorescence was recorded by excitation with a 488-nm Ar/Kr laser and detection of 500–550 nm emission fluorescence. TagRFP and MitoTracker Red fluorescence was recorded by excitation with a 543-nm He/Ne laser and detection of 580–650 nm (TagRFP) or 555–650 nm (MitoTracker Red) emission fluorescence. Images were captured by Leica Confocal software (Leica Microsystems). Images with different fluorescence channels were overlaid using Image J software (rsbweb.nih.gov/ij/), or DP Manager software (Olympus, Tokyo, Japan) when relative positions of images needed manual adjustment.

### Quantification of VHF in mature colonies

Ground mycelia were inoculated on 100-times-diluted PDA, incubated at 25°C for one to two weeks until the colony diameter reached ca. 3 cm, or after three weeks for strains with aggregated slow growth. An agar block with mycelia was cut out and stained with CFW as described above. Observation was performed on mycelial regions ca. 500 μm behind the colony periphery where VHF was found more abundantly but mycelia were sparse enough to discern individual hyphae. For quantification, two pictures with slightly different focal points were taken from each of ten fields of view, each covering an approximate area of 8067.6 μm^2^. The number of VHF events in the 2 × 10 pictures was counted and converted to the number per 1 mm^2^ to facilitate comparison to the previous report by Kayano et al [41]. Data from two independent experiments were collected and averaged.

### Quantification of conidial germination and conjugation

*E*. *bromicola* E799 mycelia were ground and spread on 100-times-diluted PDA as an inoculum. After two weeks of incubation, mycelia including conidia were scraped and suspended in 10 mL distilled water. The suspended mycelia were vortexed and double-filtered with Miracloth (EMD Biosciences, Inc., La Jolla, CA, USA). The flow-through fraction was washed twice with distilled water after centrifugation to yield conidia with little contamination by vegetative hyphae. Conidia were collected (ca. 10^6^ conidia from 10 plates with a 9 cm diameter) and resuspended at a concentration of 1.0 × 10^7^ conidia/mL in distilled water, after which 5 μL of conidial suspension was spot-inoculated on agar plates. Two different PDA concentrations (undiluted and 100x diluted) were used for inoculation since PDA is known to have an inhibitory effect on conidial fusion in the model fungus *Neurospora crassa* [49], [50]. Following the method of conidial fusion assay described previously in *N*. *crassa* [51], we quantified the rate of “germinated” conidia, defined as conidia with any hyphal protrusions, as well as the rate of conidia/conidial germlings interconnected with other conidia/conidial germlings via short hyphae.

### Transformation and protoplast fusion

Protoplast-mediated transformation was performed as described previously [40, 52] using pYHG or pYHR-Gen plasmids to obtain *Epichloë* isolates (*E*. *festucae* Fl1, E434, *E*. *typhina* E8, E1022) expressing either the H1-GFP or H1-TagRFP fusion protein. Southern blotting was performed as described previously [40] using PCR-amplified *gfp* or *tagrfp* gene fragments as probes for four isolates from each strain/plasmid combination to determine the copy number of the inserted plasmids.

Protoplast-mediated inter- and intraspecific cell fusion was performed using a method based on the transformation protocol with modifications. Protoplasts were prepared and washed according to the transformation procedures [40, 52], and were resuspended at a concentration of 1.0 × 10^8^ protoplasts/mL in STC buffer [1 M sorbitol, 50 mM Tris-Cl (pH 8.0), 50 mM CaCl_2_]. Aliquots (40 μL) of the protoplast solutions from two strains with different antibiotic markers were combined, mixed with 20 μL PEG solution [40% polyethylene glycol 4,000, 50 mM Tris-Cl (pH 8.0), 50 mM CaCl_2_, 1 M sorbitol] and incubated on ice for 30 min. This was followed by addition of 900 μL PEG solution and 20 min incubation at room temperature. Aliquots (100–400 μL) from this solution were mixed with 5 mL regeneration medium top agar (PDB containing 0.8 M sucrose and 0.8% agar, pH 6.5) and overlaid on a 15 mL regeneration plate (PDB containing 0.8 M sucrose and 1.5% agar, pH 6.5). After overnight incubation, 5 mL regeneration medium top agar containing appropriate amounts of antibiotics was overlaid on the plates to achieve the final plate concentration of 150 μg/mL hygromycin and 200 μg/mL geneticin.

For quantification of the protoplast cell fusion rate, protoplasts were generated in the lysing enzyme solution supplemented with 1% glucose to maintain the expression of fluorescent proteins for subsequent experiments. Protoplasts were washed and fused as described above. After the addition of 900 μL PEG solution and 20 min incubation at room temperature, protoplasts were checked for their fluorescence using an Olympus BX41 microscope (Olympus) equipped with a Plan N 20× objective lens (NA, 0.4; Olympus), GFP-3035B-OMF-CUST-ZERO (for GEP; 473/31 nm excitation, 520/35 nm emission, 495 nm dichroic mirror; Olympus), and TXRED-4040B-OMF-CUST-ZERO (for TagRFP; 562/40 nm excitation, 624/40 nm emission, 593 nm dichroic mirror; Olympus) filters. The number of protoplasts that had GFP fluorescence alone (a), TagRFP fluorescence alone (b), or both GFP and TagRFP fluorescence (c) was counted. The heterotypic protoplast fusion rate was subsequently calculated by the equation c/(a+b+c).

To estimate the frequency that heterotypic protoplast fusion leads to successful establishment of viable hybrid colonies, protoplast fusion solutions were inoculated on plates with or without the two antibiotics. After two weeks of incubation, the rate of putative hybrid generation was calculated as the number of colonies on selection plates containing both antibiotics, divided by the number of colonies on plates without antibiotics.

## Results

### VHF in *Epichloë festucae*

**Fig 1:**
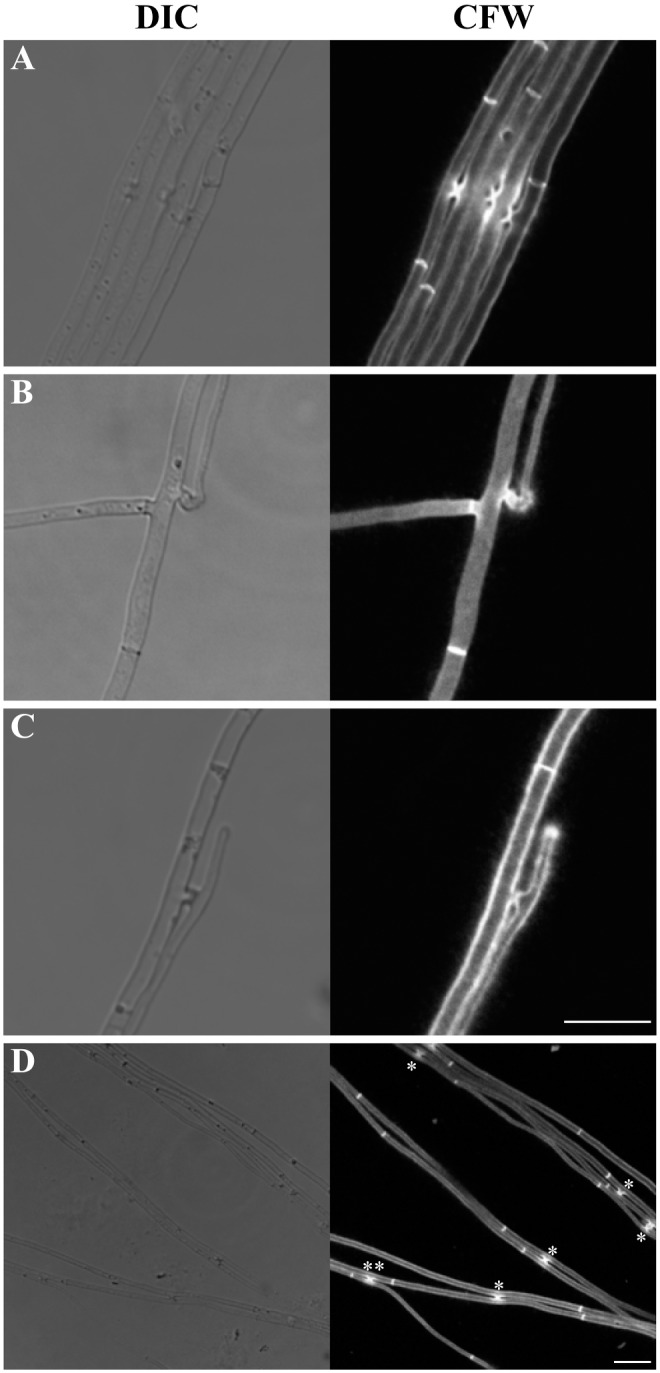
Different types of vegetative hyphal fusion in *Epichloë festucae* E2368 grown in culture. Confocal micrographs of DIC optics and Calcofluor White (CFW) staining showing lateral-to-lateral fusion **(A)**, apical-to-lateral fusion **(B)**, and subapical-to-lateral fusion **(C)**. (**D**) Though a majority of vegetative hyphal fusion occurred between two hyphae running side-by-side (asterisks), it also occurred when two hyphae came into close proximity (double asterisks). Bars represent 10 μm.

In *Epichloë festucae* E2368, a majority of VHF occurred along the lateral edges of hyphae growing parallel to one another (Fig 1A). VHF was also found where two hyphae came into close proximity (Fig 1D, double asterisks). In some cases, VHF involved hyphal apices (Fig 1B) or subapices (Fig 1C) of apical hyphal compartments. We did not observe VHF connecting two hyphae located more than 5 μm apart, suggesting the lack of long-distance hyphal chemo-attraction, as pointed out previously [41].

To investigate whether cytoplasmic connectivity and organelle redistribution followed VHF, we stained mitochondria and vacuoles of *E*. *festucae*. Mitochondria of *E*. *festucae* E2368 grown in culture were filamentous structures, which became shorter and more sparse in basal regions of the mycelium (S1A Fig), consistent with reports in other fungi [53], [54]. Vacuoles of E2368 grown in culture had a variety of sizes, with some occupying nearly an entire hyphal compartment (S1B Fig, arrow). In contrast, vacuoles of E2368 growing in the host plant typically had a more uniform size (S1C Fig). This may suggest that mature hyphae in the colony center of *E*. *festucae* grown in culture undergo autophagic degradation involving the vacuoles, as has been shown in other fungi [55], [56], whereas endophytic hyphae (*in planta*) remain metabolically active [57], [58], possibly by suppressing such vacuolar degradation. We could not assess the morphology of mitochondria *in planta*, as we were unable to stain them adequately.

**Fig 2:**
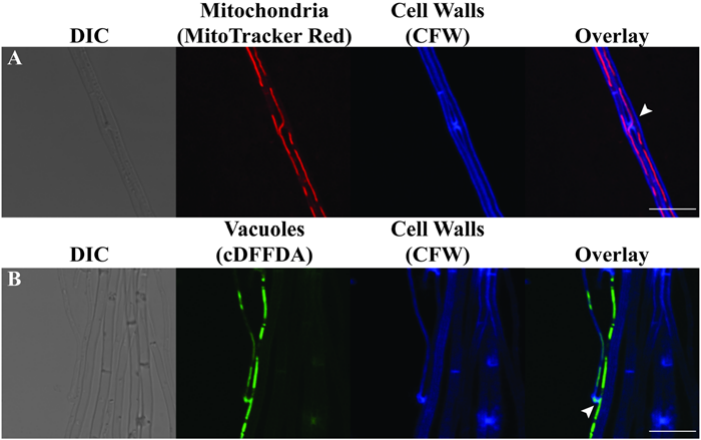
Vegetative hyphal fusion of *E*. *festucae* E2368 grown in culture establishing cytoplasmic continuity. Confocal micrographs showing DIC optics, mitochondria **(A)** or vacuoles **(B)**, CFW staining, and overlaid images. Arrowheads indicate fusion bridges through which organelles extend into a neighboring compartment. Bars represent 10 μm.

Simultaneous staining of cell walls and either mitochondria or vacuoles of mycelia in culture revealed that both organelles are capable of passing through hyphal fusion pores to a neighboring compartment (Fig 2). This indicates that cytoplasmic continuity is established by VHF to allow passage of organelles, probably along with microtubules that serve as a scaffold for these organelles [53], [59].

**Fig 3:**
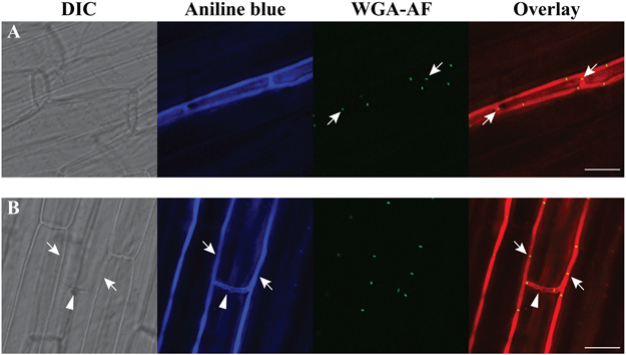
Vegetative hyphal fusion of *E*. *festucae* Fl1 in the tall fescue leaf sheath. Confocal micrographs of DIC optics, aniline blue staining showing fungal hyphae, Alexa Fluor 488-conjugated wheat germ agglutinin (WGA-AF) showing septa, and overlaid images with aniline blue staining pseudocolored in red. **(A)** Arrows point septa in two short hyphae connecting two hyphae extending in parallel. **(B)** Two hyphae (arrows) connected by a short hypha (arrowhead) which presumably underwent vegetative hyphal fusion. Locations of all three hyphae correspond to outlines of plant cells (compare DIC and aniline blue images). Bars represent 20 μm.

VHF is also found in endophytic (*in planta*) hyphae of *E*. *festucae* [27]. A clear example is shown in Fig 3A, where two hyphae running in parallel are connected by two short hyphae (arrows), indicating that at least one of these short hyphae underwent VHF. In another example shown in Fig 3B, two hyphae (arrows) are connected by a short hypha (arrowhead), with all three hyphae corresponding to the outlines of their adjacent plant cells. Note that establishment of such hyphal connection of ca. 20 μm *in planta* may not necessarily require hyphal chemo-attraction (see discussion).

### VHF in different *Epichloë* strains

Since *Epichloë* species include sexual non-hybrid species and asexual non-hybrid and hybrid species, we reasoned this is a good system to analyze the correlation among VHF, reproductive modes, and hybrid status. Therefore, we investigated frequency of VHF in forty-four different *Epichloë* strains grown in culture.

The number of VHF events was quantified in different *Epichloë* strains (Table 3), which were then categorized into three groups; those who commonly underwent VHF (++; the average number of VHF in the observed area in one set of experiments [8067.6 μm^2^] was ≥ 1, which corresponds to ca. > 10 VHF per mm^2^), those who rarely underwent VHF (+; the average number of VHF in the observed area was < 1), and those with undetectable levels of VHF (ND). We did not note any morphological difference in VHF among different strains, including the apparent lack of long-distance chemo-attraction. VHF was commonly detected in twenty-one out of twenty-six sexual non-hybrid *Epichloë* strains encompassing nine species with few exceptions (Table 3). In six *E*. *festucae* var. *lolii* (*Neotyphodium lolii*) strains, which are asexual, non-hybrid species, VHF was common in one, was rare in three, and was not detected in two strains. In twelve asexual, interspecific hybrids encompassing five species, VHF was common in six, was rare in two, and was not detected in four strains. It was also noted that the lack of VHF was not restricted to one species, and that the frequency of VHF could differ among different strains in the same species. Overall, we found that sexual *Epichloë* species are more likely to undergo VHF compared to asexual species (*P* = 0.013, Fisher’s exact test), suggesting a link between VHF and the capability to undergo sexual reproduction. The correlation between hybrid status and the frequency of VHF was less clear (*P* = 0.075, Fisher’s exact test).

Though VHF mostly occurs between two hyphae growing side-by-side (Fig 1), the likelihood of several hyphae running in parallel, as judged by the presence of “hyphal cord-like structures” (S2 Fig) did not always correlate with the degree of VHF (*P* = 0.307, Fisher’s exact test; Table 3). We also found VHF in hyphal coil structures (S3A Fig), which resemble epiphyllous coil structures shown previously [27]. Though this may support the previous idea that VHF may be important for formation of complex, three-dimensional multicellular hyphal structures [60], we also noted *Epichloë* strains forming coil structures completely devoid of hyphal fusion (S3B Fig).

### Apparent lack of conidial fusion in *Epichloë bromicola*

In many filamentous fungi, VHF also occurs at an early stage of colony development, linking conidia/conidial germlings [16], [17], [61], [62]. It has been suggested that conidial fusion, as compared to VHF in mature colonies, plays a more dominant role in fusion between genetically different individuals, which in turn may lead to horizontal transfer of genetic material [20], [63]. For this reason, we investigated whether *Epichloë* species undergo conidial fusion as well. Though *Epichloë* species are in general poor producers of conidia, we were able to harvest a sufficient amount of conidia from a strain of *E*. *bromicola* (E799). Upon inoculation on PDA or 100 times-diluted PDA, some conidia had already germinated and/or been linked with other conidia even at the time point zero. However, though the rate of germinated conidia increased significantly after 51 hrs, the rate of conidia that are linked to other conidia or conidial germlings stayed unchanged (Table 4). Thus, the presence of conidia that are linked to other conidia in *E*. *bromicola* is likely to be due to incomplete cytokinesis during conidiation rather than VHF.

**Table 4:** Rates of germinated conidia and conidia that are linked to other conidia in *E*. *bromicola* E799 grown in culture.

	0 hrs	1/100 PDA, 51 hrs	PDA, 51 hrs
Germinated conidia	55.4 ± 5.4%	94.8 ± 2.8%	95.0 ± 1.6%
Linked conidia	4.8 ± 2.1%	4.0 ± 2.4%	1.8 ± 1.3%

Average ± s. d. Two biological with four technical replicates.

### Nuclear behavior upon cell fusion in *Epichloë*

**Fig 4:**
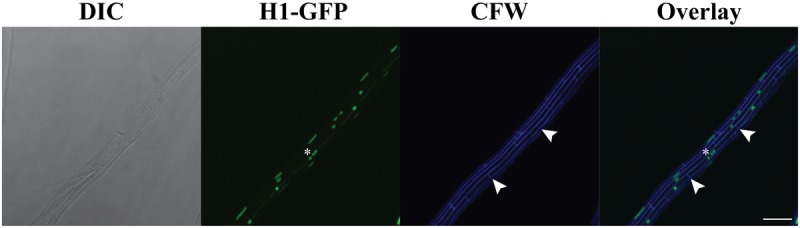
Histone H1-GFP-labeled nuclei in *E*. *festucae* Fl1 grown in culture. Nuclei in Fl1 appear as either round or elongated structures. Though most hyphal compartments are uninucleate, some compartments possess three or more nuclei (asterisk). Arrowheads indicate two septa at the end of the hyphal compartment. The bar represents 20 μm.

If VHF is to give rise to allodiploid-like hybrids, it needs to allow exchange of nuclei between fused compartments. Importantly, all *Epichloë* strains examined to date are uninucleate [58], and it is not known whether multiple nuclei can stably share the same cytoplasm, except as a transient stage during mitosis. To study nuclear behavior upon VHF, we created two plasmids that express histone H1 fused to different fluorescent proteins and harbor different antibiotic markers (plasmid pYHG for histone H1-GFP fusion with the hygromycin resistance gene and pYHR-Gen for histone H1-TagRFP fusion with the geneticin resistance gene; Table 1). We chose two *E*. *festucae* strains (Fl1, E434) and two *E*. *typhina* strains (E8, E1022) as parental strains for transformation, since these two *Epichloë* species are common parents of interspecific hybrids (e.g., see [6]), and all four strains commonly undergo VHF (Table 3). Among the obtained transformants, we chose representative isolates that had a low plasmid copy number as determined by Southern blotting, and exhibited wild-type like phenotypes. Over 90% of *Epichloë* hyphal compartments were uninucleate as expected, though there were occasional hyphal compartments in *E*. *festucae* Fl1 that had three or more nuclei (Fig 4, asterisk; Table 5).

**Table 5:** Percentages of uninucleate, binucleate, multinucleate (i.e., three or more nuclei), and anucleate hyphal compartments in *E*. *festucae* Fl1, *E*. *typhina* E1022, and the putative hybrid between Fl1 and E1022 grown in culture.

	Uninucleate	Binucleate	Multinucleate	Anucleate
Fl1	90.7 ± 4.7%	5.8 ± 3.8%	1.8 ± 0.8%	1.8 ± 1.7%
E1022	96.7 ± 0.7%	2.4 ± 0.4%	0.0%	0.9 ± 1.0%
Fl1xE1022	91.8 ± 1.4%	1.8 ± 1.0%	0.0%	6.4 ± 1.7%

Average ± S. D., three replicates of n = 150.

**Fig 5:**
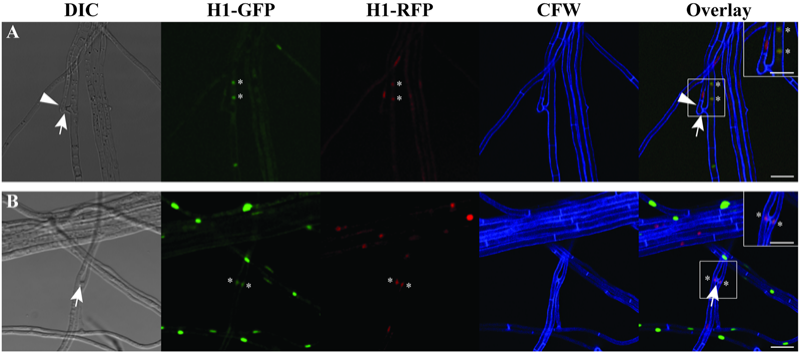
Vegetative hyphal fusion between two *Epichloë* transformants grown in culture expressing either GFP- or TagRFP-fused histone H1. **(A)** Vegetative hyphal fusion (arrow) between two *E*. *festucae* Fl1 transformants resulted in an anucleate hyphal compartment (arrowhead) and a binucleate compartment with the two nuclei (asterisks) possessing both GFP and RFP signals. **(B)** Vegetative hyphal fusion (arrow) between hyphae of *E*. *festucae* E434 expressing histone H1-GFP and *E*. *festucae* Fl1 expressing histone H1-TagRFP, resulting in nuclei (asterisks) in each compartment having both GFP and RFP signals. Bars in large pictures represent 10 μm, whereas those in insets are 5 μm.

To observe nuclear behavior upon VHF, two *Epichloë* transformants expressing different fluorescent proteins fused to histone H1 were co-cultured. In Fig 5A showing VHF between two *E*. *festucae* Fl1 transformants expressing either H1-GFP or H1-TagRFP, two hyphal compartments had fused (arrow), resulting in an anucleate (left, arrowhead) and a binucleate compartment (right, asterisks), with the two nuclei in the latter appearing yellow due to the co-existence of both GFP and TagRFP signals. It should be noted that the presence of “yellow” nuclei does not necessarily indicate nuclear fusion and subsequent division, since it could be a result of exchanging histones between the two nuclei through the shared cytoplasm, as noted previously [20].

When two alternative *Epichloë* isolates were co-cultured, VHF between hyphae of the two was much rarer, apparently due to different hyphal growth patterns which tended to keep the hyphae of the two strains separate rather than in close proximity. In the example of Fig 5B showing fusion between *E*. *festucae* E434 expressing H1-GFP and *E*. *festucae* Fl1 expressing H1-TagRFP, two nuclei remained in their respective compartments with both nuclei appearing yellow, suggesting exchange of fluorescently labeled histones. Thus, these results indicate VHF allows two types of nuclei to stably share the same cytoplasm long enough for “yellow nuclei” to emerge by either exchange of histones or nuclear fusion followed by nuclear division.

To test whether VHF leads to emergence of hybrid *Epichloë*, co-cultures of transformants of two distinct *Epichloë* species, each having a different antibiotic resistance marker, were subcultured on PDA containing both antibiotics. However, the obtained mycelia were always a mixture of the two parental strains in which two types of fluorescence resided in separate hyphae, rather than a hybrid. As an alternative approach, we performed protoplast fusion experiments, followed by selection of resulting colonies on plates containing both antibiotics. After protoplast fusion between *E*. *festucae* Fl1 expressing H1-GFP and *E*. *typhina* E1022 expressing H1-TagRFP, 11.2 ± 3.2% (average ± s. d., three biological with three technical replicates) of protoplasts with nuclear fluorescence represented heterotypically fused protoplasts, which simultaneously had GFP and TagRFP fluorescence. Although this result indicated that 11.2% of protoplasts were double-resistant to the antibiotics, the number of colonies that emerged on double-selection plates was only 0.062% ± 0.061% (average ± s. d., three biological with two technical replicates) of the number of colonies on non-selection plates. Thus, it appeared that only a tiny fraction of heterotypically fused protoplasts successfully established colonies on selection plates.

**Fig 6:**
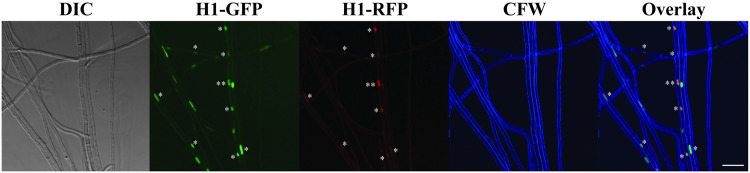
Nuclear distribution in a putative hybrid grown in culture. The putative hybrid was generated through a protoplast fusion experiment between *E*. *festucae* Fl1 expressing histone H1-GFP and *E*. *typhina* E1022 expressing histone H1-TagRFP. Most hyphal compartments are uninucleate, with nuclei possessing both GFP and TagRFP signals (asterisks) without any vegetative hyphal fusion nearby. The bar represents 20 μm.

The putative hybrids that emerged on double-selection plates grew with growth rates and colony morphology comparable to both or either of parental strains. Confocal microscopy revealed that the putative hybrids mostly possessed hyphal cells containing both GFP and TagRFP fluorescence within the same nucleus without any obvious VHF nearby, even though TagRFP fluorescence was somewhat weaker (Fig 6). Importantly, in all putative hybrids, hyphal compartments were predominantly uninucleate (Table 5), and we did not observe multinucleate mycelia, which would be expected for heterokaryotic strains. Collectively, these results strongly suggest that H1-GFP- and H1-TagRFP-encoding genes, as well as the two antibiotic resistance genes, coexist within the same nuclei in the putative hybrids, presumably as a result of horizontal gene/chromosome transfer or nuclear fusion.

## Discussion

### Cell biological features of VHF in *Epichloë*

Similar to that observed in other filamentous fungi [64], VHF in *Epichloë* species establishes cytoplasmic continuity [41], and allows exchange of cytoplasmic organelles including mitochondria, vacuoles (Fig 2) and probably nuclei as well (Fig 5A). One notable difference is the apparent lack of long-distance chemo-attraction to facilitate interaction between two hyphae ([41], Fig 1). In soil-dwelling saprotrophic fungi, which may grow freely in three-dimensional space, two hyphae may be unlikely to meet by chance without chemo-attraction. In contrast, the natural growth pattern of *Epichloë* are confined to that laid down by the plant cells. As a hyphal branch grows laterally between plant cells, it is likely to meet another hypha eventually without chemo-attraction (e.g., see Fig 3B). VHF may occur once the branch comes close enough to the other hypha, as we observed in culture (Fig 1D), accounting for long hyphal connection of ca. 20 μm that we observed *in planta* (Fig 3B).

Another notable difference in VHF of *Epichloë* is an apparent lack of conidial fusion. However, this may not be surprising considering the physiological role of conidia in *Epichloë*. The primary role of *Epichloë* conidia is to serve as spermatia for sexual reproduction, which gives rise to ascospores that play a major role in horizontal dissemination and colony initiation by infecting a new host [24], [25]. Though conidia are also produced during the asexual cycle from epiphyllous hyphae [23], [27] and may play a role in dissemination [65], they are in general sparse [25] and are not likely to provide a large density of conidia, which seems to be a prerequisite for conidial fusion [62]. We note however, the recent suggestion that *Epichloë* conidia produced at the sexual reproduction stage also play a role in dissemination [66], though it is not known whether they result in a large density of conidia upon colony initiation.

It is already known that *Epichloë* species lack a vegetative incompatibility system [21]. Vegetative incompatibility describes a mechanism leading to programmed cell death of fused compartments after VHF between two incompatible fungal individuals [19]. This phenomenon is believed to have evolved as a mechanism preventing potentially deleterious outcomes of fusion with “non-self” individuals, such as the acquisition of somatic parasites and viruses. As discussed by Chung and Schardl [21], in contrast to saprotrophic fungi that share their habitats with other fungi, *Epichloë* are normally isolated within individual host plants and host seeds, and are unlikely to encounter other fungal individuals, since only one *Epichloë* strain infects one plant [67], [68]. Thus, the lack of vegetative incompatibility probably represents infrequent occurrence of VHF between different *Epichloë* strains in nature.

### Roles of VHF in *Epichloë*

The physiological roles of VHF in *Epichloë* species remain unclear. Previous studies showed that mutant *E*. *festucae* strains lacking VHF are unable to establish mutualistic symbiosis, and lead to the death of the host plant [40–43]. However it has also been discussed that these fatal effects on the host plant may be due to other functions of these genes besides those involved in VHF [40].

Our analysis of VHF in different strains revealed a clear trend that sexual *Epichloë* species are more likely to undergo fusion, suggesting a potential link between the ability to undergo sexual reproduction and VHF. *Epichloë* species have a bipolar, heterothallic mating system in which perithecium development requires transmission of spermatia (conidia) into protoperithecia of the opposite mating type [69]. It is possible that proteins required for VHF also play roles in perithecium development, as has been reported in other fungi [17]. Conversely, the lower frequency of fusion in asexual strains may suggest that hyphal fusion is less important during the vegetative stage of *Epichloë*. By allowing exchange and distribution of water, nutrients, and signaling molecules [16–18], VHF is probably of particular importance in saprotrophic fungi, where different mycelial regions encounter heterogeneous microenvironments with different levels/types of nutrient resources [70]. In contrast, the apoplastic environment occupied by these endophytes may be more homogeneous and hyphae may have access to similar levels of nutrients/water. Theoretically, this could make exchange of resources between distinct mycelial regions less important.

### Nuclear behavior following VHF and protoplast cell fusion of *Epichloë*

In order for cell fusion to give rise to transfer of genetic material, two types of nuclei need to share the same cytoplasm. Such cohabitation of nuclei may readily occur in many filamentous fungi by heterokaryon formation following fusion [20], [51], [71]. Though *Epichloë* species are typically uninucleate fungi, we demonstrated that two types of nuclei can share the same cytoplasm after VHF (Fig 5). This contrasts with another uninucleate fungus, *Fusarium oxysporum*, in which VHF is immediately followed by migration of one nucleus into the neighboring fused cell, and subsequent degradation of the resident nucleus [72].

Previous protoplast fusion experiments using multinucleate fungi led to relatively high rates (1–10% for compatible fusion) of colonies on selection plates [73–75]. These colonies were almost exclusively heterokaryons in which different sets of chromosomes from each parent coexist within the same cytoplasm (or mycelium) in separate nuclei (e.g., [73], [76]). In contrast, our experiments using *Epichloë* species resulted in a much lower colony recovery rate (0.06%), with all of resulting colonies appearing to be uninucleate. The simultaneous presence of both markers (i.e., GFP and TagRFP) in hyphal compartments (Fig 6) strongly suggests that the single nuclei contain both marker genes, unlike heterokaryons in other fungi described above. A strong possibility is that the uninucleate nature of *Epichloë* species precludes a prolonged multinucleate heterokaryotic status and restricts the number of fused protoplasts that survive to mature colonies. If this is indeed the case, it would suggest that there is a rather severe bottleneck, with hybrid *Epichloë* species emerging only when nuclear fusion rapidly follows VHF. In either case, an essential next step for this study is to uncover the genomic composition of the putative hybrids, especially their ploidy and how the two marker genes are maintained in cells.

### Does VHF give rise to hybridization in *Epichloë*?

Though putative hybrids were readily generated from protoplast cell fusion, we emphasize caution in relating this mechanistically to hybridization events of *Epichloë* species in nature. For example, interspecific cell fusion at the sexual reproduction stage, rather than the vegetative stage, might lead to hybridization. However, mating barriers are generally strong in *Epichloë* species [6], [77], suggesting that interspecific mating is unlikely to occur. Further, *Epichloë* species, which exhibit a bipolar, heterothallic mating system, require strains of opposite mating types for fertilization [69]. Intriguingly, it has recently been reported that several interspecific hybrid *Epichloë* species have two copies of the same mating type idiomorph [78–80], strongly suggesting that they are not the result of sexual hybridization. Thus VHF remains the most likely explanation for hybridization in *Epichloë* species (see also [6], [38]).

If the preceding argument holds, it follows that when VHF between two *Epichloë* strains does occur, the fused compartments could stay alive due to the lack of vegetative incompatibility [21]. Subsequently, the two types of nuclei could stably coexist within the same cytoplasm (Fig 5), potentially followed by nuclear fusion and the formation of allodiploid-like hybrids. However, the relatively rare encounter of two strains [21] and the low rate of successful colony establishment after cell fusion (as shown for protoplast fusion) suggest that emergence of hybrid *Epichloë* genotypes is exceedingly rare compared to other fungi. How then to explain the recognized prevalence of hybrid *Epichloë*? For example, from the 59 endophytes isolated by Moon et al. [35] and Gentile et al. [36], 44 were hybrids [38], whereas hybrids in other fungi are very rare [22], [81] with only a limited number of known examples [5], [6], [82]. This apparent discrepancy suggests that the prevalence of *Epichloë* hybrids is due to post-hybridization selection, rather than the frequent occurrence of hybridization. It has been shown that fitness disadvantages of hybrids, if there are any, are magnified during intense competition with their non-hybrid relatives, and lead to elimination of hybrid genotypes from the population [83]. Indeed, since fungal hybrids typically have fitness disadvantages such as sterility [7] and a bigger genome size [7], [8], [34], they would have only a small chance of survival unless they avoid such competition. In *Epichloë* species, a newly emerged hybrid will experience a short period of competition with its non-hybrid relatives; however, once it successfully infects a new host individual, there will be no more competition since typically only one *Epichloë* strain infects one plant [67], [68]. In addition, hybridization may allow infection of a new host species [6], [38], further reducing competition. Without competition, fitness disadvantages of hybrids including sterility and a bigger genome size are unlikely to be major problems. What is probably more important is the fitness of the host plant that may be enhanced by a new combination of protective traits that the hybrid provides [37], which in turn allows hybrid *Epichloë* to flourish. Thus, the low competition that hybrids experience, along with fitness increases experienced by both the host plant and *Epichloë* may explain the prevalence of *Epichloë* hybrids.

Though hybridization of *Epichloë* may be infrequent in nature, protoplast fusion readily gave rise to putative hybrids with marker genes from two parents. This suggests the possibility of creating non-genetically modified hybrids by using methods for selecting fused protoplasts devoid of artificial marker genes (e.g., [84], [85]). If our assumption of low competition and fitness increase allowing survival of hybrid *Epichloë* is correct, generated hybrids will be sufficiently sustainable for practical use. We suggest that protoplast fusion followed by a large-scale screening of putative hybrids will be an important next step towards improving agronomic potential of *Epichloë* species.

## Supporting Information

S1 Fig Mitochondria and vacuoles of *E*. *festucae* E2368 grown in culture. (A) Mitochondria stained with MitoTracker Red. From left, long and dense mitochondria in apical regions, shorter mitochondria in subapical regions, sparse and round mitochondria in basal regions. (B) Vacuoles stained with cDFFDA. The arrow points a hyphal compartment nearly entirely occupied by a large vacuole. (C) Vacuoles in hyphae growing in the tall fescue leaf sheath stained with cDFFDA. Bars represent 20 μm.
(PDF)

S2 Fig Hyphal cord-like structures in *Epichloë* grown in culture. DIC optics and Calcofluor White (CFW) staining showing examples of hyphal cord-like structures of *Epichloë* endophytes. Bars represent 20 μm.
(PDF)

S3 Fig Calcofluor White staining showing hyphal coil structures. CFW staining showing examples of hyphal coil-like structures. Hyphal fusion (white arrow) is seen in the hyphal coil of *E*. *typhina* subsp. *clarkii* E426, whereas the hyphal coil of *E*. *elymi* E757 is devoid of hyphal fusion, with spiral hyphae aligned in an orderly manner. The left image is from a single focal plane, while the right is a maximum projection image from a z-series. Bar represents 20 μm.
(PDF)
