# Supplementary material for: Vegetative Hyphal Fusion and Subsequent Nuclear Behavior in Epichloë Grass Endophytes
Source: PLoS One. 2015 Apr 2;10(4):e0121875. doi: 10.1371/journal.pone.0121875 (PMC4383479; doi:10.1371/journal.pone.0121875)
Supplement: S1 Fig — (A) Mitochondria stained with MitoTracker Red. From left, long and dense mitochondria in apical regions, shorter mitochondria in subapical regions, sparse and round mitochondria in basal regions. (B) Vacuoles stained with cDFFDA. The arrow points a hyphal compartment nearly entirely occupied by a large vacuole. (C) Vacuoles in hyphae growing in the tall fescue leaf sheath stained with cDFFDA. Bars represent 20 μm. (PDF) [file pone.0121875.s001.pdf]

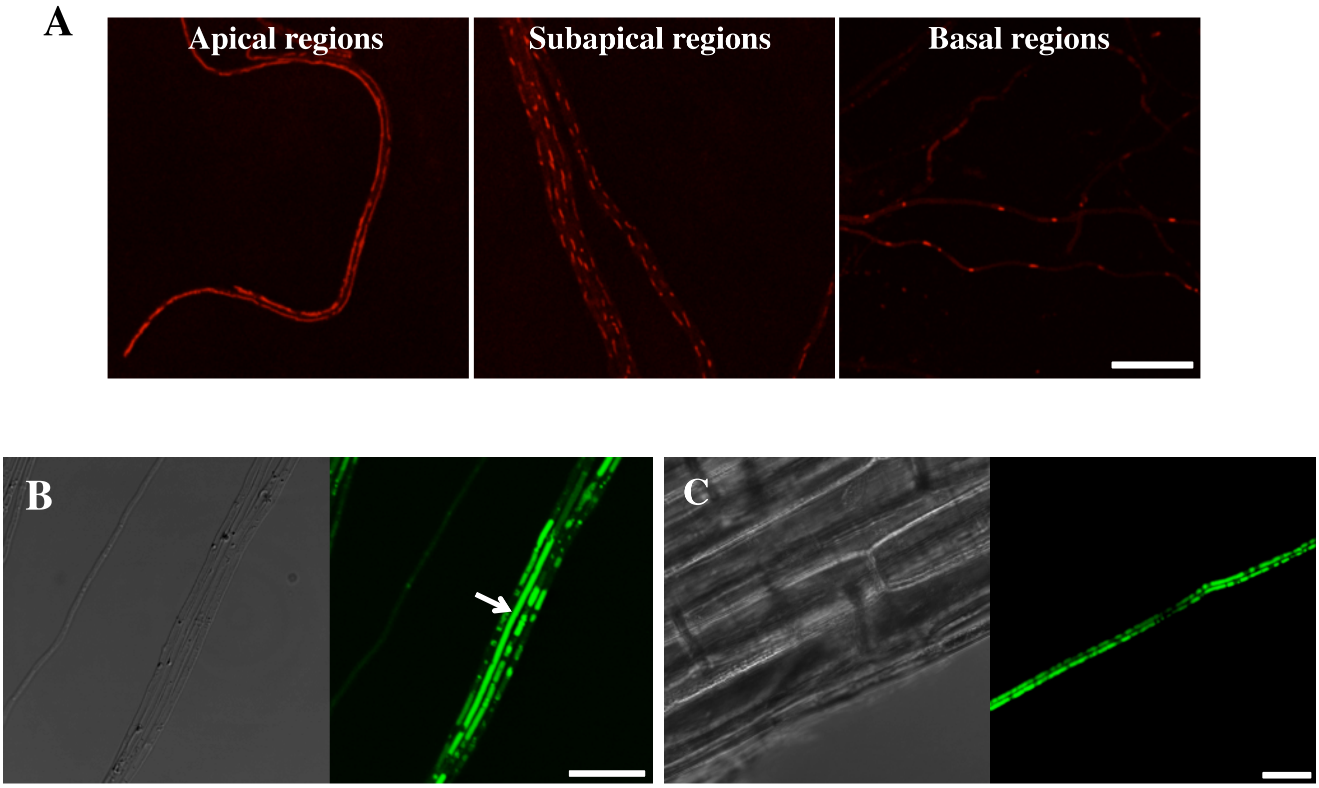

S1 Fig. Mitochondria and vacuoles of *E. festucae* E2368 grown in culture. (A) Mitochondria stained with MitoTracker Red. From left, long and dense mitochondria in apical regions, shorter mitochondria in subapical regions, sparse and round mitochondria in basal regions. (B) Vacuoles stained with cDFFDA. The arrow points a hyphal compartment nearly entirely occupied by a large vacuole. (C) Vacuoles in hyphae growing in the tall fescue leaf sheath stained with cDFFDA. Bars represent 20 μm.

Extensive hyphal cords	Minor hyphal cords	No hyphal cords
(*E. amarillans* E57)	(*Epichloë* sp PauTG-1 e55)	(*E. festucae* E1035.33)

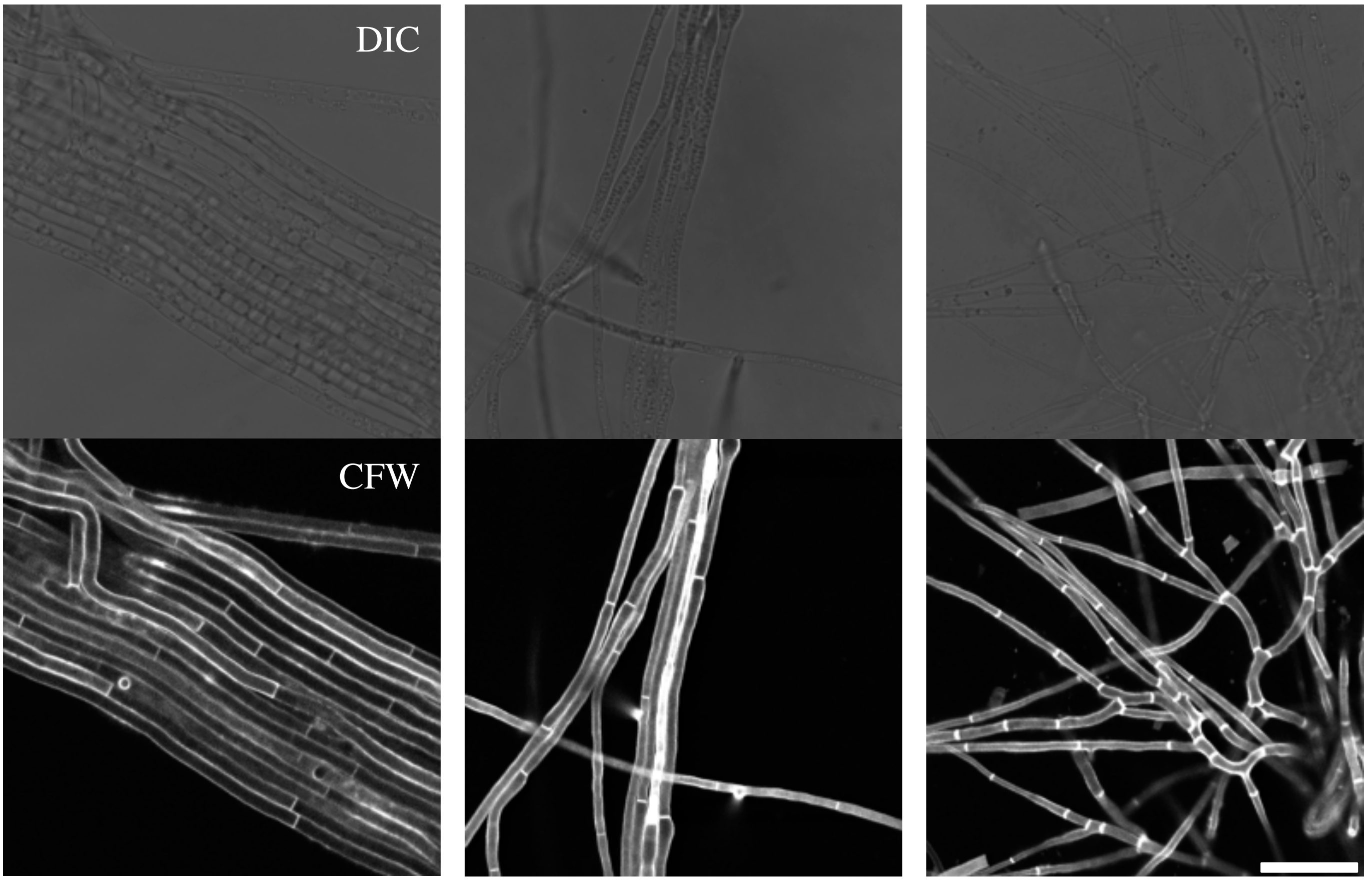

S2 Fig. Hyphal cord-like structures in *Epichloë* grown in culture. DIC optics and Calcofluor White (CFW) staining showing examples of hyphal cord-like structures of *Epichloë* endophytes. Bars represent 20 μm.
