# Supplementary material for: Vegetative Hyphal Fusion and Subsequent Nuclear Behavior in Epichloë Grass Endophytes
Source: PLoS One. 2015 Apr 2;10(4):e0121875. doi: 10.1371/journal.pone.0121875 (PMC4383479; doi:10.1371/journal.pone.0121875)
Supplement: S2 Fig — DIC optics and Calcofluor White (CFW) staining showing examples of hyphal cord-like structures of Epichloë endophytes. Bars represent 20 μm. (PDF) [file pone.0121875.s002.pdf]

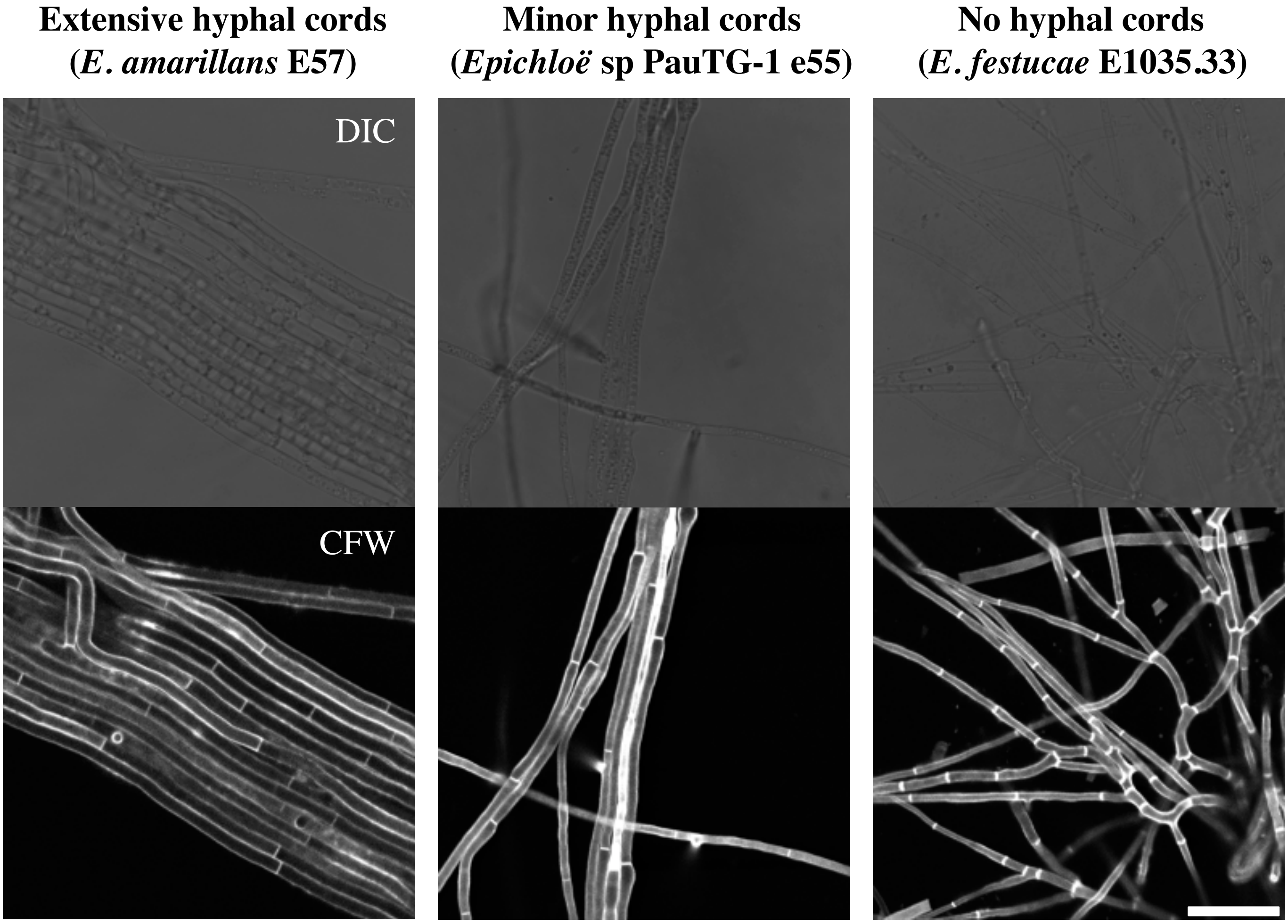

S2 Fig. Hyphal cord-like structures in *Epichloë* grown in culture. DIC optics and Calcofluor White (CFW) staining showing examples of hyphal cord-like structures of *Epichloë* endophytes. Bars represent 20 μm.
