# Supplementary material for: Vegetative Hyphal Fusion and Subsequent Nuclear Behavior in Epichloë Grass Endophytes
Source: PLoS One. 2015 Apr 2;10(4):e0121875. doi: 10.1371/journal.pone.0121875 (PMC4383479; doi:10.1371/journal.pone.0121875)
Supplement: S3 Fig — CFW staining showing examples of hyphal coil-like structures. Hyphal fusion (white arrow) is seen in the hyphal coil of E. typhina subsp. clarkii E426, whereas the hyphal coil of E. elymi E757 is devoid of hyphal fusion, with spiral hyphae aligned in an orderly manner. The left image is from a single focal plane, while the right is a maximum projection image from a z-series. Bar represents 20 μm. (PDF) [file pone.0121875.s003.pdf]

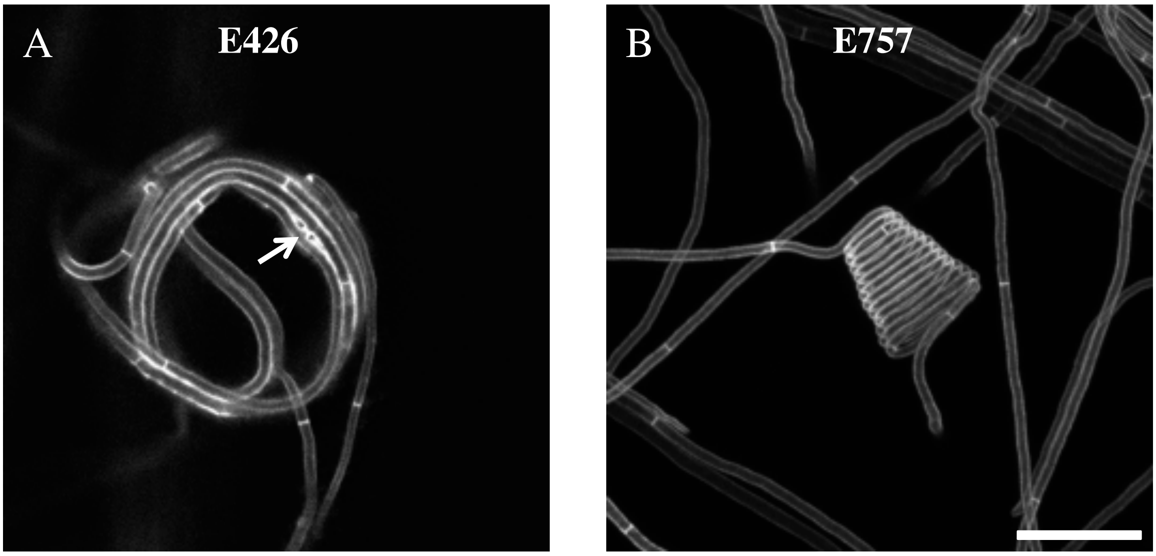

S3 Fig. Calcofluor White staining showing hyphal coil structures. CFW staining showing examples of hyphal coil-like structures. Hyphal fusion (white arrow) is seen in the hyphal coil of *E. typhina* subsp. *clarkii* E426, whereas the hyphal coil of *E. elymi* E757 is devoid of hyphal fusion, with spiral hyphae aligned in an orderly manner. The left image is from a single focal plane, while the right is a maximum projection image from a z-series. Bar represents 20 μm.
